# Semiautomated 3D mapping of aneurysmal wall enhancement with 7T-MRI

**DOI:** 10.1038/s41598-021-97727-0

**Published:** 2021-09-15

**Authors:** Ashrita Raghuram, Alberto Varon, Jorge A. Roa, Daizo Ishii, Yongjun Lu, Madhavan L. Raghavan, Chaorong Wu, Vincent A. Magnotta, David M. Hasan, Timothy R. Koscik, Edgar A. Samaniego

**Affiliations:** 1 Department of Neurology, University of Iowa Carver College of Medicine, 200 Hawkins Drive, Iowa City, IA 52246 USA; 2 Department of Neurosurgery, University of Iowa Carver College of Medicine, Iowa City, IA USA; 3 Roy J Carver Department of Biomedical Engineering, University of Iowa, Iowa City, IA USA; 4 Institute for Clinical and Translational Science, University of Iowa, Iowa City, IA USA; 5 Department of Radiology, University of Iowa Carver College of Medicine, Iowa City, IA USA; 6 Department of Psychiatry, University of Iowa Carver College of Medicine, Iowa City, IA USA

**Keywords:** Cerebrovascular disorders, Neurovascular disorders, Predictive markers, Brain imaging, Magnetic resonance imaging

## Abstract

Aneurysm wall enhancement (AWE) after the administration of contrast gadolinium is a potential biomarker of unstable intracranial aneurysms. While most studies determine AWE subjectively, this study comprehensively quantified AWE in 3D imaging using a semi-automated method. Thirty patients with 33 unruptured intracranial aneurysms prospectively underwent high-resolution imaging with 7T-MRI. The signal intensity (SI) of the aneurysm wall was mapped and normalized to the pituitary stalk (PS) and corpus callosum (CC). The CC proved to be a more reliable normalizing structure in detecting contrast enhancement (p < 0.0001). 3D-heatmaps and histogram analysis of AWE were used to generate the following metrics: specific aneurysm wall enhancement (SAWE), general aneurysm wall enhancement (GAWE) and focal aneurysm wall enhancement (FAWE). GAWE was more accurate in detecting known morphological determinants of aneurysm instability such as size ≥ 7 mm (p = 0.049), size ratio (p = 0.01) and aspect ratio (p = 0.002). SAWE and FAWE were aneurysm specific metrics used to characterize enhancement patterns within the aneurysm wall and the distribution of enhancement along the aneurysm. Blebs were easily identified on 3D-heatmaps and were more enhancing than aneurysm sacs (p = 0.0017). 3D-AWE mapping may be a powerful objective tool in characterizing different biological processes of the aneurysm wall.

## Introduction

**Figure 1:**
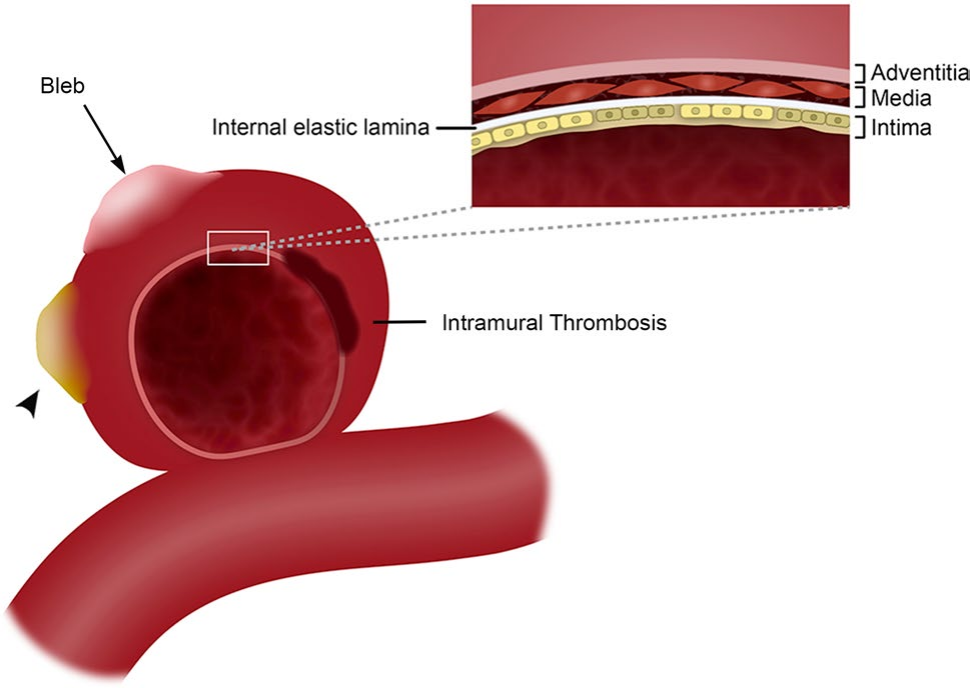
Vessel wall layers of an intracranial aneurysm with a bleb (arrow) and atherosclerotic calcification (arrowhead). The aneurysm wall has a complex morphology with multiple biological processes. This image belongs to the author Edgar A Samaniego.

High resolution-magnetic resonance imaging (HR-MRI) provides a high contrast-to-noise ratio and spatial resolution for better visualization of intracranial arterial walls. Biological processes such as microhemorrhages, wall thickening, atherosclerotic changes or indirect signs of inflammation may be visualized with HR-MRI. Within this spectrum of radiological findings, aneurysmal wall enhancement (AWE) after the administration of gadolinium (Gd) has been described as a marker of aneurysm instability^1^. The characterization and understanding of AWE as a surrogate marker of changes in aneurysm wall biology is a nascent field. Histological analysis of aneurysm walls shows endothelial disruption as a potential marker of aneurysm instability^2^. Formation of atherosclerotic lesions as a result of lipid accumulation is one example of the complex remodeling mechanisms that affect the aneurysm walls^2,3^. Histological analysis has linked Gd uptake in the aneurysm wall to intimal disruptions in the internal elastic lamina and endothelial cells^4,5^. AWE may be used as a biomarker of instability once the dynamic contrast uptake by different layers of the aneurysm wall is better characterized (Fig. 1).

Meta-analysis and several reports have consistently shown that AWE is significantly correlated with higher risk of aneurysm growth and rupture^6^. The definitions of AWE vary broadly, although recently there has been a step forward in determining AWE objectively instead of subjectively^7–9^. One of the major limitations in the current method of assessing AWE is that images are processed and analyzed in multiplanar 2D views and in the best-case scenario, include different segments of the aneurysm. This approach is limited by the manual sampling performed by the investigator in determining which areas of the aneurysm have increased AWE.

There is also no consensus in determining which areas of the aneurysm wall should be analyzed. Detailed analysis of focal AWE (FAWE) has determined that the colocalization of these areas with low wall shear stress, such as blebs (Fig. 1) is associated with a higher risk of rupture^10,11^. Other groups determined a higher risk of rupture when circumferential wall enhancement (CAWE) is present, instead of FAWE^1,12,13^. Intramural thrombosis (Fig. 1) has also been associated with increased CAWE^14^. These different approaches in analyzing AWE are aimed at identifying aneurysms prone to grow and rupture. However, these approaches have the inherent limitation of using manual 2D multiplanar sampling to characterize a 3D structure with complex morphology such as brain aneurysms. Aneurysm size and morphological indices such as size and aspect ratios have been correlated with risk of rupture^15–18^. These indices may be used in validating new methods of AWE characterization.

In this study we describe a semi-automated method of generating 3D-AWE maps of the entire aneurysm. The main aim of this approach is to characterize different biological processes that affect the aneurysm wall and may manifest with different patterns of AWE. We believe that a comprehensive 3D analysis of the aneurysm will lead to a better estimation of the risk of rupture in prospective clinical studies.

## Results

Thirty patients with 33 unruptured intracranial aneurysms (UIAs) were included in the analysis: 28 saccular and 5 fusiform aneurysms. The average aneurysm spoke length based on wall thickness was 0.55 ± 0.09 mm and the average surface area/aneurysm was 311.8 ± 680 mm^2^. An average of 62 spokes per mm^2^ were generated. Only saccular aneurysms were included in the morphological analysis (Table 1) as it has been described that fusiform aneurysms undergo different biological processes^19^. The entire cohort was included in validation of the AWE semi-automated method.

**Table 1:** Characteristics of saccular aneurysms.

Variable	Total aneurysms N = 28	GAWE (+) N = 7	GAWE (−) N = 21	*p*
**Demographic**				
Age (mean ± SD, years)	66 ± 9.1	72 ± 8.8	64 ± 8.5	**0.04**
Men (%)	8 (29)	1 (14)	7 (33)	0.33
Hypertension (%)	18 (64)	6 (86)	12 (57)	0.17
Hyperlipidemia (%)	13 (46)	3 (43)	10 (48)	0.83
Smoking (%)	6 (21)	0	6 (29)	0.11
**Morphological parameters**				
Diameter (mean ± SD, mm)	8.2 ± 6.56	12.39 ± 8.13	6.8 ± 5.5	**0.049**
Size ratio (mean ± SD)	2.51 ± 1.78	3.95 ± 2.59	2.02 ± 1.12	**0.010**
Aspect ratio (mean ± SD)	2.12 ± 1.61	3.63 ± 2.35	1.61 ± 0.89	**0.002**
Ellipticity index (mean ± SD)	0.24 ± 0.03	0.24 ± 0.02	0.22 ± 0.03	0.2
Non-sphericity index (mean ± SD)	0.28 ± 0.05	0.31 ± 0.04	0.27 ± 0.05	0.13
Undulation index (mean ± SD)	0.15 ± 0.06	0.16 ± 0.06	0.14 ± 0.6	0.48
Surface area (mean ± SD, mm^2^)	311.8 ± 680.4	617.6 ± 852.5	209.8 ± 602.7	0.17
Volume (mean ± SD, mm^3^)	913 ± 2729.1	1769.5 ± 3198.6	627.5 ± 2577.4	0.35
Wall thickness (mean ± SD, mm)	0.55 ± 0.09	0.62 ± 0.1	0.51 ± .09	**0.008**
Bleb (%)	14 (50)	3 (43)	11 (52)	0.66
Mural thrombosis (%)	4 (14)	3 (43)	1 (4)	**0.013**
**Location**				
ICA (%)	12 (43)	3 (43)	9 (43)	0.163
ACOM (%)	7 (25)	1 (14)	6 (29)	
Basilar (%)	3 (11)	1 (14)	2 (10)	
MCA (%)	4 (14)	0	4 (19)	
PICA (%)	1 (4)	1 (14)	0	
PCOM (%)	1 (4)	1 (14)	0	
**Aneurysm wall enhancement (AWE)**				
Δ CC ratio (mean ± SD)^a^	0.85 ± 0.24	0.56 ± 0.15	0.24 ± 0.12	**0.001**
Percent increase (mean ± SD)^a^	63 ± 41	96 ± 48	52 ± 0.33	**0.012**

^a^T1 to T1 + Gd. Bold values indicate a significance less than 0.05.

### Measurement of AWE and selection of normalizing structure

**Figure 2:**
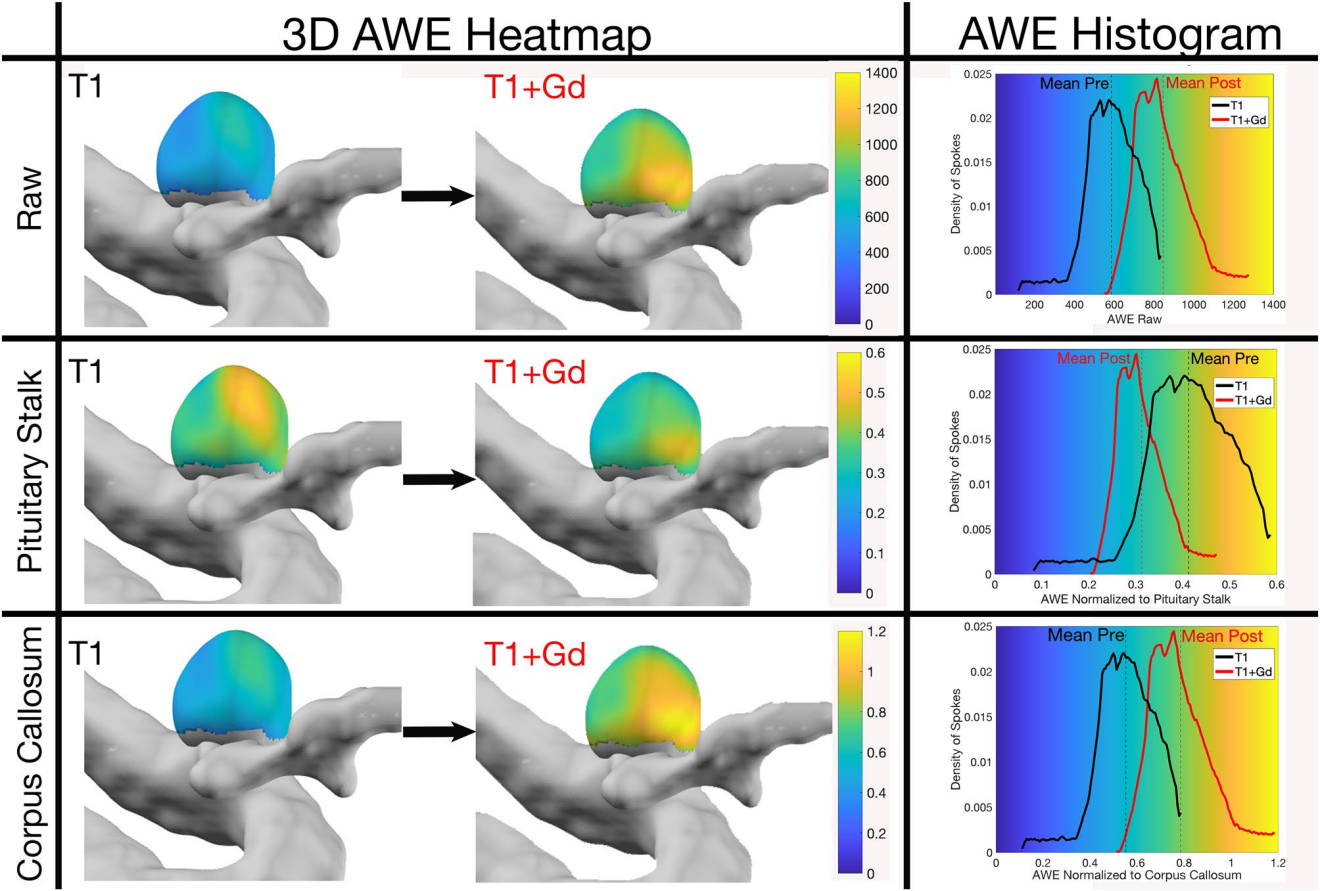
3D heatmaps of T1 and T1 + Gd AWE of a PICA aneurysm. Histograms were generated from 3D maps: y axis = spoke density and x axis = AWE raw values (first row) and AWE ratios for the PS (second row) and corpus callosum (third row). The corpus callosum was a better structure for normalization and had an almost identical curve when compared to raw values (first and third rows). Avid PS Gd enhancement shifts the ratio to the left in T1 + Gd images (middle row). This aneurysm is highly enhancing as show in T1 + Gd images. *AWE* aneurysm wall enhancement, *PICA* posterior inferior cerebellar artery.

**Figure 3:**
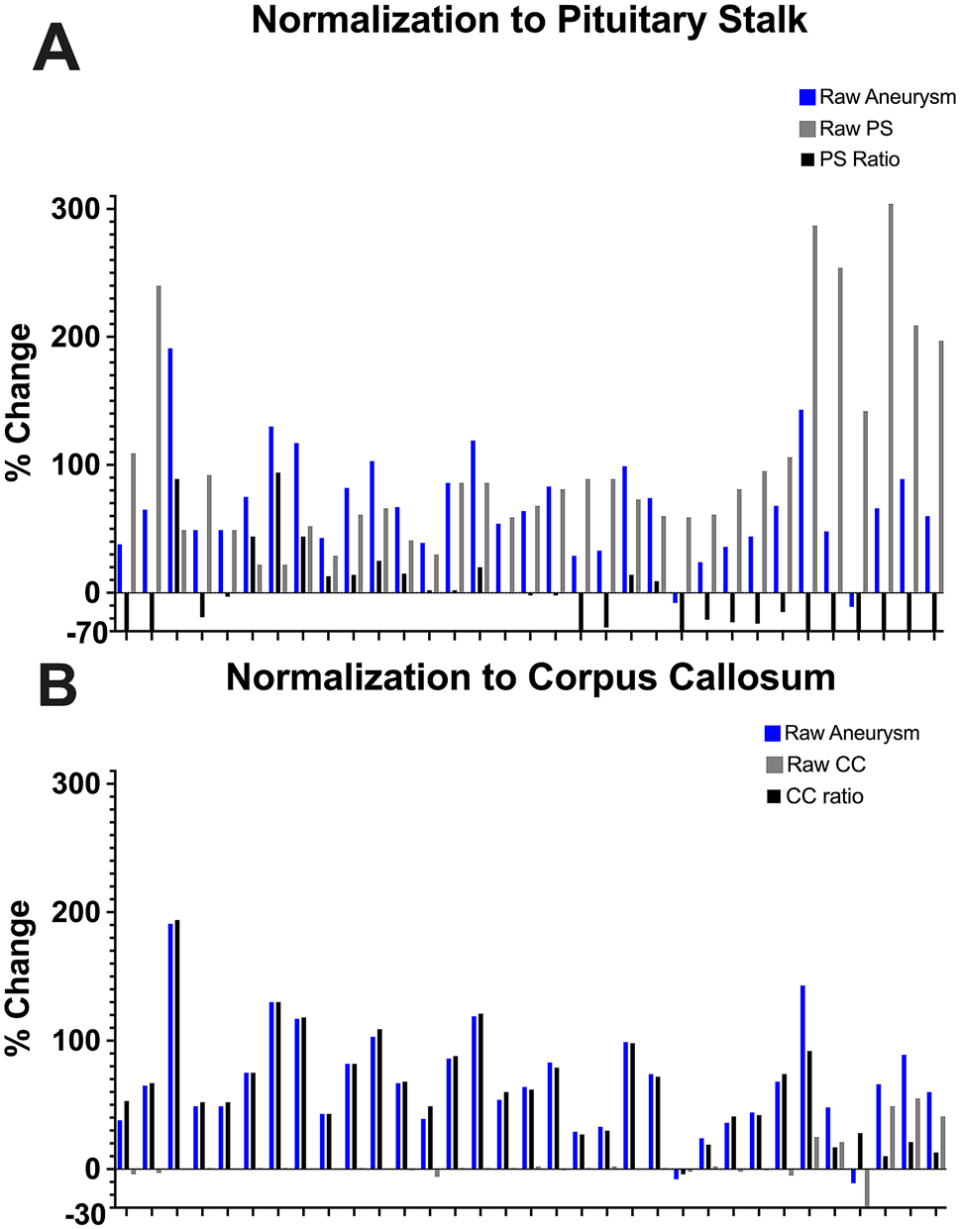
Percentage change (T1 vs T1 + Gd) in AWE without any normalization (blue) is compared to the percentage change in normalizing structures (grey) and the normalized ratio (black = blue/grey). The pituitary stalk uptakes avidly Gd and generates artifact (**A**, blue vs grey). In contrast, raw AWE mirrors the Gd uptake of the CC, rendering a more reliable ratio (**B**). *CC* corpus callosum.

**Figure 4:**
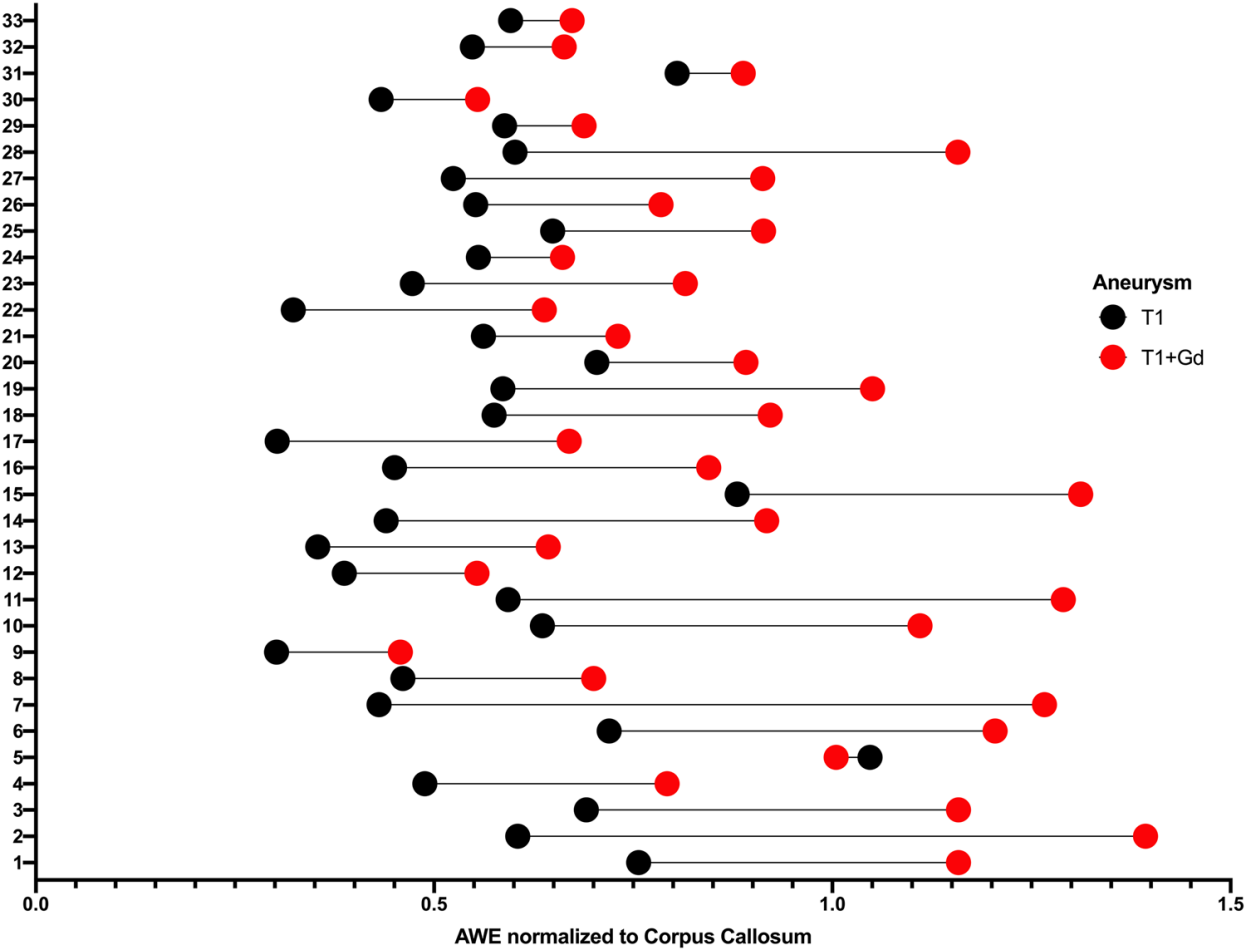
Whisker plot representation of each aneurysm demonstrating the change in AWE normalized to the CC between the pre (black) and the post-Gd (red) T1 sequences. T1 AWE normalized to CC µ = 0.56 ± 0.16. T1 + Gd AWE normalized to CC µ = 0.89 ± 0.25. There is a wide range of Gd uptake suggesting that aneurysms walls are very heterogeneous. *AWE* aneurysmal wall enhancement.

Comparisons between manual and semi-automated methods showed a correlation for CC and PS ratios (r = 0.792, r = 0.806, p < 0.001). Histogram analysis showed that normalization to the CC provided a more reliable shift of the curve from lower AWE in T1 sequences towards higher AWE in T1 + Gd sequences. In some cases, avid enhancement of the PS introduced artifact and the curve shifted to the left (Fig. 2). As a result of this artifact, 58% of PS ratios were negative (T1 + Gd < T1) versus 3% of CC ratios. AWE displayed as the shift of CC ratio from T1 to T1 + Gd was also significant (p ≤ 0.0001), as opposed to the PS ratio (p = 0.10) (Fig. 3, Table 2). The coefficient of variation of the CC was lower (28%) than the PS (38%) (Table 2). Therefore, despite previous data^7^, the CC appears to be a more reliable normalization structure in determining AWE (∆ T1 + Gd − T1) within the same aneurysm (Fig. 4).

**Table 2:** Distribution of enhancement ratios of the PS and CC for aneurysms and blebs.

	T1		T1 + Gd		% Change from pre to post
	Mean ± SD	Coefficient of variation (%)	Mean ± SD	Coefficient of variation (%)	
**Aneurysm**					
Pituitary stalk	0.482 ± 0.178	36.89	0.424 ± 0.159	37.73	− 12.04% (p = 0.1)
Corpus callosum	0.564 ± 0.165	29.18	0.892 ± 0.25	28.06	58% (p** < 0.0001)**
**Bleb**					
Pituitary stalk	0.474 ± 0.152	32.06	0.427 ± 0.202	47.2	− 9.92% (p = 0.28)
Corpus callosum	0.548 ± 0.151	27.6	0.905 ± 0.319	35.28	65.15% (p < **0.0001)**

Bold values indicate significance less than 0.05.

### Histogram analysis and definitions of AWE

**Figure 5:**
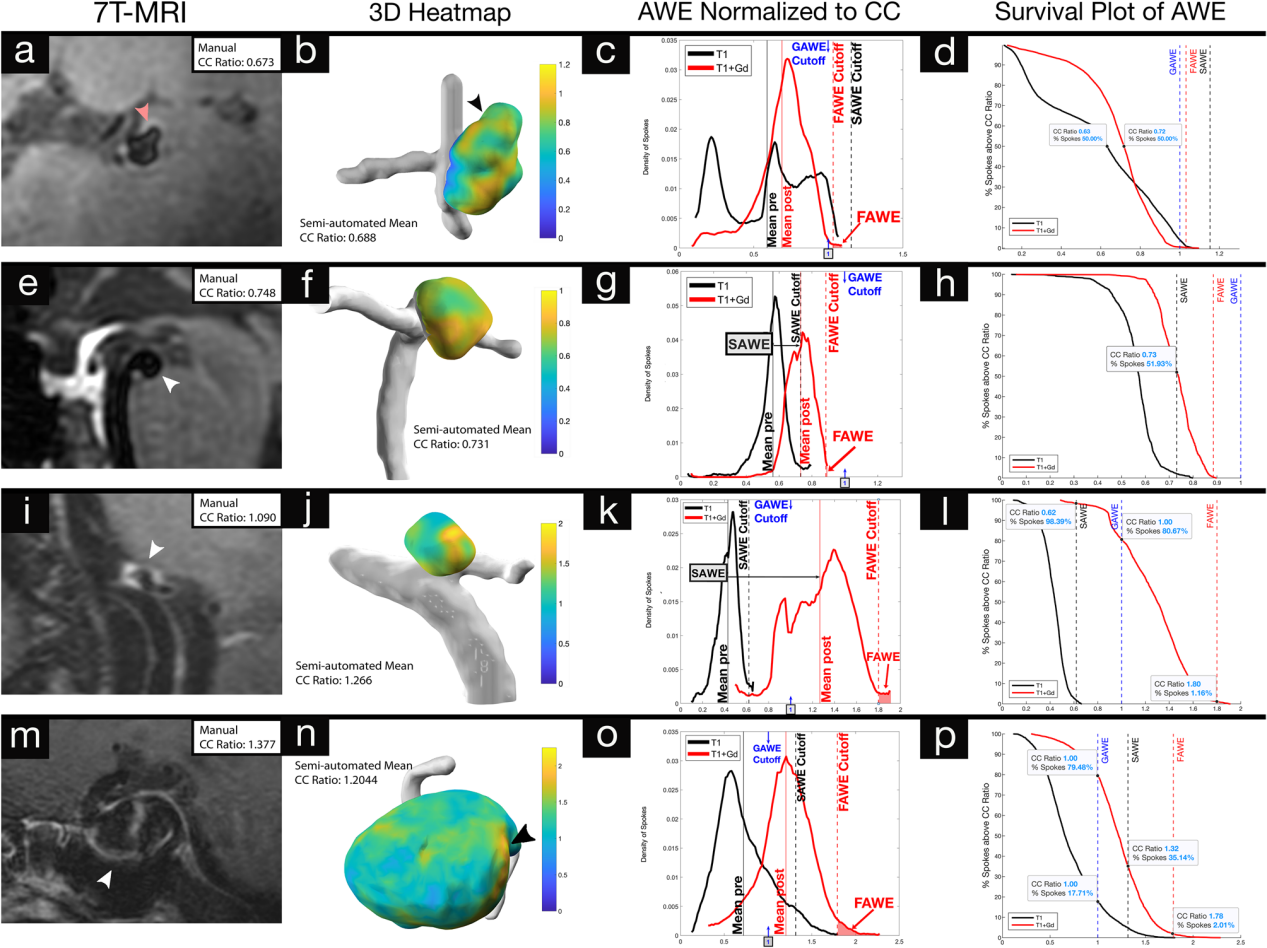
3D-Heatmaps generated from 7T-MRI T1 + Gd images provide comprehensive topographic views of AWE while histograms and survival plots quantify dynamic changes in AWE before and after contrast administration: specific to each aneurysm (SAWE), against generalized thresholds for enhancement (GAWE) and identifies regions of focal AWE (FAWE). (**a**–**d**) An M2 aneurysm with a daughter sac (red arrowhead) appears enhancing on 2D-views (**a**). The 3D-Heatmap (**b**) shows heterogeneity in AWE. Histogram analysis (**c**) establishes thresholds for SAWE based on pre-contrast AWE distribution, GAWE (CC ratio ≥ 1, blue arrow) and FAWE based on post-contrast AWE distribution. 50% of spokes had a CC ratio > 0.72 in the T1 + Gd reconstruction compared to 0.63 in the T1 reconstruction, indicating a marginal level of enhancement that does not meet the defined criteria for SAWE or GAWE (**d**). This suggests pseudo-AWE seen on the MRI (**a**), which may be related to slow flow in this distal M2 bifurcation aneurysm. (**e**–**h**) This basilar tip aneurysm does not appear enhancing despite its location being associated with a high risk of rupture (**e**). The 3D-Heatmap (**f**) shows enhancement. Analysis of the histogram (**g**) identifies the aneurysm as SAWE+. The majority of spokes (~ 52%) lie above the SAWE threshold (**h**). This aneurysm does not exhibit a large FAWE area (**g**). (**i**–**l**) PICA aneurysm with documented growth appears highly enhancing on the MRI (**i**). FAWE visualized on the 3D-Heatmap (**j**) is identified on the histogram (**k**). Histogram analysis indicates that this aneurysm is both SAWE + (black arrow) and GAWE + (blue arrow). (**l**) Indicates that > 90% of spokes lie above both thresholds, and that ~ 1% of spokes lie above the FAWE threshold. Despite documented growth, this aneurysm has benign morphological features associated with a low risk of rupture (size = 3.8 mm, AR = 1.76). (**m**–**p**) This large ICA aneurysm appears highly enhancing on MRI (**m**) and a strong area of FAWE (arrowhead) is evident on the 3D-heatmap (**n**). Histogram analysis (**o**) identifies the aneurysm as GAWE+ and SAWE-. Some parts of the aneurysm (17%) met the criteria for GAWE before contrast administration, and only ~ 30% of spokes lie above the SAWE threshold (**p**). *3D-Heatmaps and histograms were generated on aneurysm specific scales to better visualize the enhancement topography.

For each aneurysm, the distribution of spokes was plotted according to SI ratios of T1 and T1 + Gd images. Three metrics of AWE were determined: (1) Specific AWE (SAWE), defined as the change in enhancement between T1 and T1 + Gd for each aneurysm (T1 + Gd µ ≥ T1 µ + 2σ). (2) General AWE (GAWE), defined as the average CC ratio of all SAWE + aneurysms (µ = 1.0023) or CC Ratio ≥ 1. GAWE was used to compare AWE between different aneurysms. (3) Focal AWE (FAWE), defined as areas of high AWE with two SDs above the mean of the T1 + Gd distribution (≥ T1 + Gd µ + 2σ) (Fig. 5). Survival plots were used to measure the percentage of spokes of each category of enhancement.

### SAWE, GAWE and FAWE analysis

Eleven aneurysms were SAWE+ and had a higher percentage increase in AWE from T1 to T1 + Gd (μ = 88%), when compared to non-enhancing aneurysms (μ = 47%, p = 0.008). Moreover, enhancing aneurysms had a higher increase in CC ratio (μ = 0.46) from T1 to T1 + Gd vs non-enhancing aneurysms (μ = 0.22, p < 0.001). The average CC ratio for enhancing SAWE+ aneurysms was 1.002 (p = 0.001). Seven aneurysms were GAWE+ and had a higher percentage increase in CC ratio from T1 to T1 + Gd (μ = 96.3%) vs non-enhancing aneurysms (μ = 52%, p = 0.012). Enhancing GAWE+ aneurysms also had a higher CC ratio increase (μ =  + 0.56) between T1 and T1 + Gd when compared to non-enhancing aneurysms (μ =  + 0.24, p < 0.001) (Table 1). Twenty-three aneurysms (82%) displayed FAWE. Of these aneurysms, 15 (65%) had only one area of FAWE. Aneurysms that had more than one area of focal enhancement (n = 8, 35%) had a larger percentage of spokes above the FAWE cutoff (μ = 3.5%, p = 0.005). Histograms of aneurysms with more than one area of FAWE had a positive skew (μ = 0.315, p = 0.04). Histograms of aneurysms that had only one area of FAWE had a negative skew (μ = − 0.430, p = 0.04).

### 3D morphological analysis

**Figure 6:**
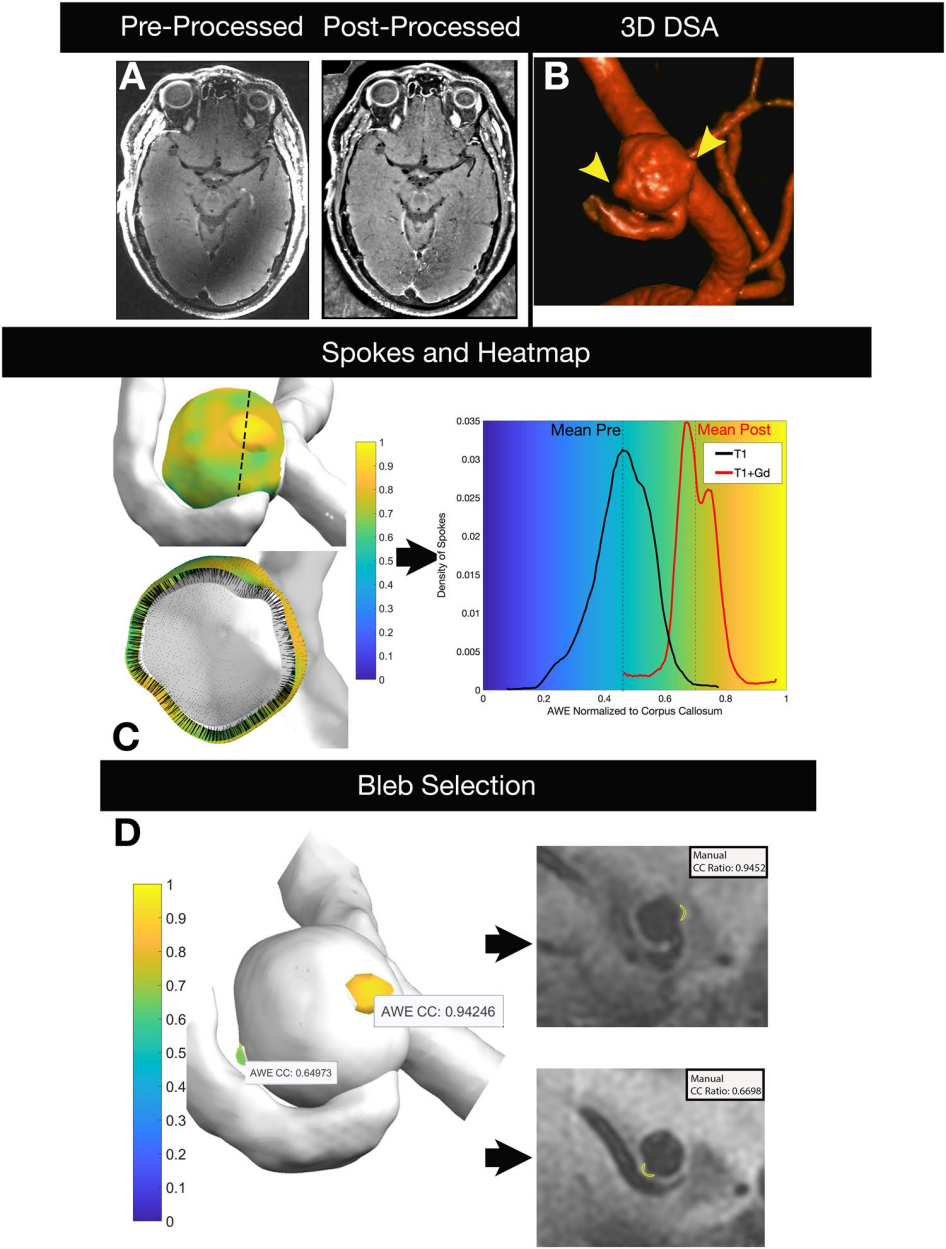
High-resolution images are generated from 7T-MRI and processed to optimize co-registration, decrease artifact and facilitate masking. The 3D rotational angiogram demonstrates an ACOM aneurysm with two blebs (arrowheads). Spokes are projected from the inner lumen to the outer boundary of the aneurysm wall and are used to generate a 3D heatmap (center row). The length of each spoke is tailored to the thickness of the aneurysm wall. AWE is normalized to the CC in T1 and T1 + Gd images and is used to generate the histogram that matches the 3D heatmap on the same scale. Blebs were isolated (bottom left) and AWE values were compared against manual ROIs drawn on 2D images (bottom right). There was a high correlation between values generated by the semi-automated and manual methods. Blebs, otherwise not seen on 2D multiplanar imaging, were easily visualized and analyzed on 3D heatmaps. *ACOM* anterior communicating.

**Figure 7:**
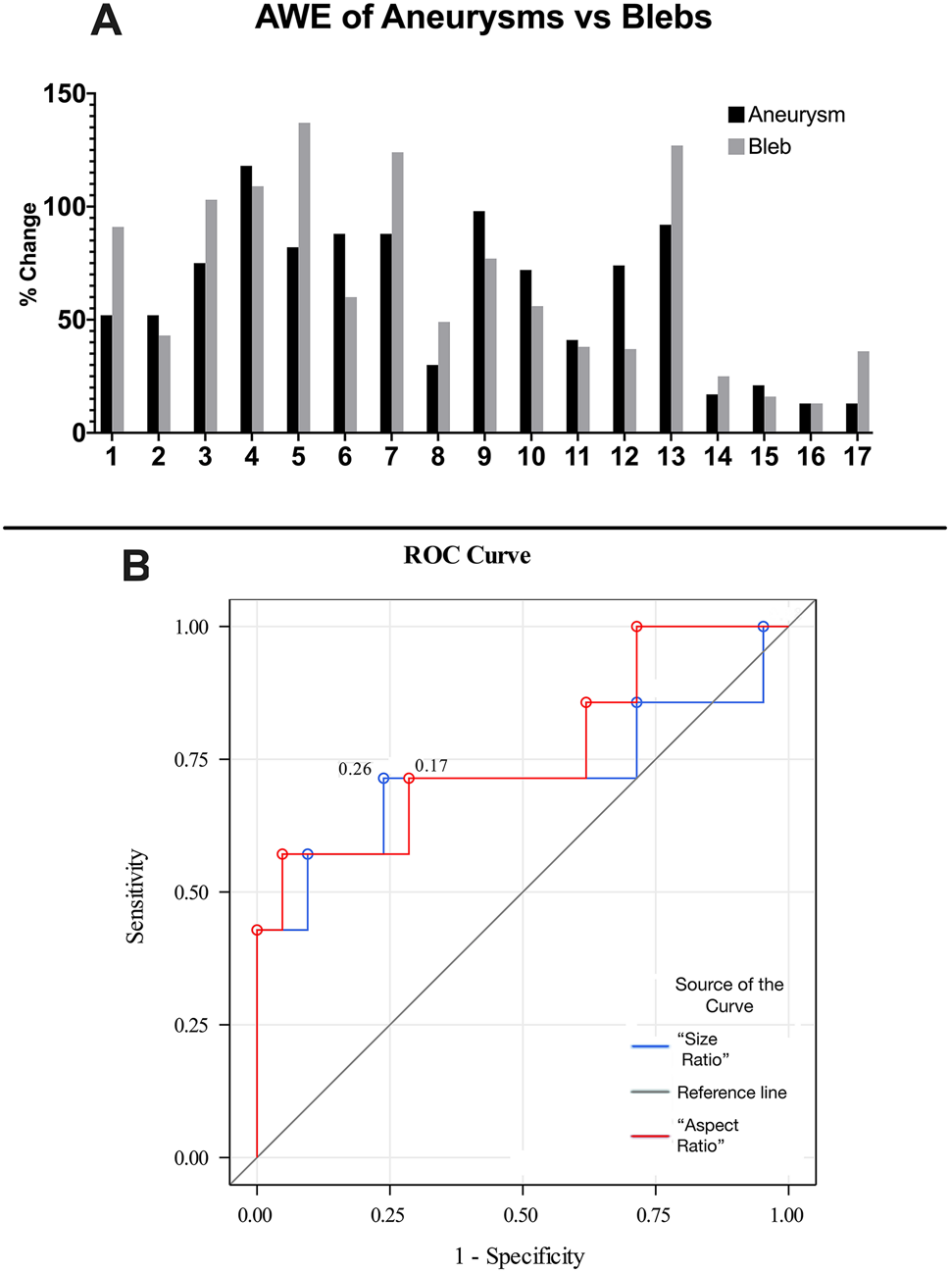
(**A**) Gd uptake of the aneurysm sac and its bleb (CC ratio ≥ 1). Fourteen aneurysms had blebs, and three aneurysms had two blebs (1 & 2, 6 & 7, 16 & 17). Gd bleb uptake varied within the same aneurysm. Overall, blebs exhibited more enhancement than sacs. (**B**) GAWE ROC analysis (CC ratio ≥ 1). Aspect ratio ≥ 3.56 (specificity 95%, sensitivity 57%) and size ratio ≥ 2.89 (specificity 76%, sensitivity 71%) best predicted GAWE + aneurysms. *GAWE* general aneurysmal wall enhancement.

3D-AWE maps showed the same morphology as depicted in 3D rotational angiograms (Fig. 6). This allowed a separate and detailed analysis of the aneurysmal sac versus blebs. The maximal AWE was significantly different between the sac and blebs (p = 0.0017). Nine of 17 aneurysm (53%) with blebs exhibited increased AWE in the bleb, compared to the sac, mean AWE increased 8% (Fig. 7A). Two-tailed Student’s t-tests of morphological characteristics demonstrated that GAWE+ aneurysms had a thicker wall (0.62 ± 0.1 mm) than non-enhancing aneurysms (0.51 ± 0.09 mm, p < 0.008). Aneurysm size (12.39 ± 8.13 mm, p = 0.049), size (3.95 ± 2.59, p = 0.01) and aspect ratios (3.63 ± 2.35, p = 0.002) were also significantly different between GAWE ± aneurysms (Table 1).

Bivariable logistic regression analysis of aneurysms ≥ 7 mm in diameter showed as predictors of instability: size ≥ 2.16 (OR 23.6, 95% CI 2–278) and aspect ratios ≥ 2.31 (OR 7.88, 95% CI 1–45). These morphological parameters have also been identified by other groups as reliable indicators of instability^20,21^. In establishing AWE as a CC ratio with a threshold ≥ 1 (GAWE+), bivariate analysis showed significance for aspect (OR 2.23, 95% CI 1–4) and size ratios (OR 1.90, 95% CI 1–3). In ROC analysis, aspect ratio ≥ 3.56 (specificity 95%, sensitivity 57%) and size ratio ≥ 2.89 (specificity 76%, sensitivity 71%) best predicted GAWE + aneurysms (Fig. 7B). Aneurysm size, size and aspect ratios were measured from DSA (24 aneurysms, 73%), CTA (5, 15%), and MRA (4, 12%). The two-way random intraclass correlation coefficients for manual measurements of aneurysm size, size and aspect ratios, and wall thickness were 0.94 (p < 0.001), 0.90 (p < 0.001), 0.63 (p < 0.001) and 0.70 (p < 0.001) respectively. Bland–Altman plots for these measurements are provided in Supplementary Figs. S1–S4.

## Discussion

This study performed a detailed topographic analysis of AWE using images acquired with 7T HR-MRI. We developed a protocol to generate and analyze 3D-AWE maps of the entire aneurysm. With this tool new metrics could be objectively analyzed and used to better understand the distribution of AWE along the aneurysm wall.

The generation of 3D color maps of AWE may become a powerful tool in quantifying enhancement and better understanding aneurysm biology. Khan et al. analyzed 25 aneurysms and subjectively determined the presence of partial or complete enhancement coupled with hemodynamic simulation and morphological measurement for instability analysis^22^. Isonormals were used to measure AWE normalized to the nominal intensity of the image volume. This innovative approach fell short of adding objective parameters of determining AWE. In this study we generated thousands of datapoints (µ = 4490 spokes/aneurysm) from orthogonal spokes projected into the aneurysm wall axis. The measurement of AWE was optimized with 7T HR-MRI by tailoring the spoke length to the thickness of the aneurysm wall for each aneurysm (range 0.38–0.78 mm). We believe that this customized sampling of AWE decreases artifact introduced by low and high enhancing structures such as the cerebrospinal fluid and the cavernous sinus, respectively. Kleinloog et al. demonstrated on 7T MRI that the radiological aneurysm wall thickness can vary between 0.2 and 1.6 mm^23^. A tailored spoke length generates exceptionally reliable 3D maps of AWE. This approach has several advantages over previously described methods of AWE analysis: 3D data allows identification of areas of aneurysm instability such as blebs, the aneurysm is visualized and analyzed in its whole magnitude, and histograms generated through this protocol provide a new tool in studying the biology of brain aneurysms and in identifying possible markers of instability. Meta-analysis and preliminary prospective data have shown that AWE is a potential predictor of aneurysms instability^8,24,25^. However, there is no consensus on how to determine thresholds of AWE or what part of the aneurysm should be sampled to analyze enhancement. 3D maps may also provide further information about the aneurysm biology as it has been shown that aneurysm compartments have different patterns of enhancement directly influenced by local flow conditions^10^. 7T MRI studies have demonstrated a linear correlation between wall thickness and SI. Histological analysis of aneurysm walls has shown an eight-fold variation in thickness^23^. Different biological processes encompass this wide variation in thickness and structure of the aneurysm wall^2^. Atherosclerotic and non-atherosclerotic calcification^26^, inflammation^27^, wall remodeling with mural necrosis^28^, proliferation of vasa-vasorum^29^, among other changes, will determine the “health” of the aneurysm wall and risk of rupture^30^ (Fig. 1). A 3D-AWE map analysis may provide a comprehensive insight into the heterogenous processes that lead to aneurysm growth and rupture.

Assessment of AWE has been performed subjectively by most groups, which limits reproducibility and affects accuracy. Even the “objective methods” of AWE analysis introduce subjectivity since ROIs are manually drawn to select areas of the aneurysm deemed as “enhancing”^7,9^. To standardize the analysis of AWE, we previously analyzed multiple enhancement metrics and determined that a PS ratio ≥ 0.60 had a sensitivity of 81% in detecting aneurysms ≥ 7 mm in diameter. This threshold correlated well with clinical predictive scales of aneurysm growth and rupture^31^. However, AWE analysis was performed in multiplanar reconstructions at best and did not capture the 3D structure of the aneurysm. This objective metric for AWE, provided a framework for our objective 3D analysis. We determined a strong correlation between our new 3D method and manual multiplanar analysis (r = 0.792). Our previous studies suggested that the PS is a reliable normalizing structure. However, in this study the PS exhibited an avid uptake of Gd in T1 + Gd images, which is suboptimal when analyzing specific AWE (SAWE). Other studies have successfully used white matter structures for normalization of enhancement through pre/post Gd indices^9,13,32^. After multiple comparisons and detailed histogram analysis, normalization to the CC resulted in a more reliable parameter in determining AWE. (Fig. 2).

The generation of AWE histograms provided a new set of metrics for analysis. Specific thresholds were determined based on histogram analysis: general AWE (GAWE), specific AWE (SAWE), and focal AWE (FAWE). Coupling this data to 3D reconstructions allowed qualitative and quantitative assessments of AWE (Fig. 6). Interestingly, aneurysms with no morphological parameters suggestive of instability, but with documented growth, were both GAWE and SAWE positive, with > 90% of spokes with CC ratios above both thresholds (Fig. 5i–l). The threshold for SAWE was determined as a shift of CC ratio of at least two SDs from the mean enhancement on T1 images. This approach allowed the analysis of Gd uptake by each aneurysm. An aneurysm-based analysis would be required in determining risk of rupture in patients with multiple aneurysms. GAWE was defined as a CC ratio ≥ 1 on T1 + Gd images. This threshold was used to compare different aneurysms. Some aneurysms that were GAWE+, did not meet the threshold for SAWE, because they had some enhancement on T1 pre imaging (Fig. 5m–p). Other aneurysms that were GAWE- and did not appear enhancing on MRI, were SAWE and FAWE positive (Fig. 5e–h). Qi et al. used pharmacokinetic modeling to estimate the contrast extravasation rate (K^trans^) in identifying aneurysms that ruptured over time. This appeared to be a more reliable metric than the analysis of enhancement determined by T1 + Gd images only^33^. SAWE may be a better tool in detecting “leaky” aneurysms when compared to GAWE. SAWE provides a map of enhancement for each aneurysm, which is an individualized metric inherent to Gd uptake in each segment of the wall. Moreover, SAWE has the potential of controlling for enhancement artifacts such as slow-flow^34^, as histogram analysis of T1 and T1 + Gd images has the potential of identifying pseudoenhancement (Fig. 5a–d).

The generation of AWE heatmaps allowed the identification of areas of increased enhancement including blebs. Larsen et al. have shown that areas of focal enhancement have low wall-shear stress conditions that may favor growth and rupture^11^. 3D-AWE maps ease identification of these areas of focal enhancement which on conventional 2D imaging may not be detected. An objective threshold for FAWE was determined by detailed histogram and 3D map analysis. The highest SI region in the histogram could identify both the threshold for FAWE and whether there are multiple areas of FAWE in the aneurysm sac (μ = 0.035, p = 0.005). Aneurysms with larger areas of FAWE also had histograms with positive skew, suggesting that these highly enhancing focal areas can affect the shape of the histogram. FAWE has been linked to intraluminal thrombus and sites of rupture^35^, this analysis could further identify and quantify these areas. 3D-AWE maps showed that on average, blebs exhibited 8% higher enhancement than the aneurysm sac. Further topographic analysis may lead to the identification of bleb-prone areas in the aneurysm sac.

This study is limited by the sample size and the heterogeneity of aneurysms included (15% fusiform). However, the study was aimed at developing a protocol for 3D-AWE mapping and not at determining specific differences between aneurysms subtypes. With this aim in mind, we used a 7T platform that eased the identification and analysis of the aneurysm wall. We are cognizant of the limited clinical use of 7T-MRI and we plan in optimizing this protocol for 3T MRIs. The nature of the small and heterogenous sample limits the predictive value of AWE metrics studied. A larger analysis using 3T-MRI is needed to determine the clinical relevance of such methods. 7T HR-MRI poses its own challenges limiting patient enrollment. Additionally, a lack of advanced blood suppression techniques may have resulted in flow artifact as a potential source of noise. While the goal of this technique is to map the aneurysm wall, the contributions of flow artifact to AWE cannot be ruled out. However, as demonstrated on Fig. 5a–d, histogram analysis could be used in detecting flow artifact.

To summarize, we believe that 3D-AWE mapping may be a powerful tool in studying the biology of brain aneurysms and in identifying aneurysms that may grow and rupture. This method generates a new set of metrics which could potentially be correlated with different biological processes of the aneurysm wall.

## Materials and methods

### Image acquisition and processing

After approval by the University of Iowa Institutional Review Board, patients with UIAs underwent HR-MRI on a GE 7T MRI (Discovery MR950) from August 2018 to December 2020. All patients provided an informed consent to participate in the study. All methods were completed in accordance with relevant guidelines and regulations. T1-weighted (sagittal CUBE 3D acquisition, 332 slices, matrix = 324 × 512 × 512, FOV = 256 × 256 mm, voxel size = 0.5 × 0.5 × 1 mm, slice spacing = 0.5 mm, TR = 0.7 s, TE = 0.01041 s, Echo Train Length = 22, flip angle = 90°) and T2-weighted images (sagittal CUBE 3D acquisition,166 slices, matrix = 162 × 256 × 256, FOV = 256 × 256 mm, voxel size = 1 × 1 × 1 mm, slice spacing = 1 mm, TR = 2.5 s, TE = 0.071837 s, Echo Train Length = 100, flip angle = 90°) were acquired. T1-weighted images were acquired before and five minutes after administering Gd. All image processing was completed with Advanced Normalization Tools^36^ and the FMRIB Software Library^37^, as well as custom scripts. DICOM datasets were converted to NIfTI-1 format for pre-processing. All images were denoised using a Rician distribution model^38^. T1 images were aligned to a template brain to approximate ACPC alignment. T2 and T1 post-Gd were then co-registered using rigid, affine, and symmetric normalization to the aligned T1 pre contrast image. Intensity non-homogeneity in T1 was corrected using three sequential procedures: the T1/T2 method^39,40^, the N4 method^41^, and the 3dUnifize function in the AFNI toolbox^42^ as well as custom scripts.

### Structure labeling and measurement

Masks of each aneurysm were generated with 3D Slicer^43^ on post-processed T1 and T1 + Gd images. Binary label maps were then created and resampled to the base image. Additionally, the genu of the corpus callosum (CC) was marked as a 3 mm spherical region of interest (ROI). Another ROI was selected in the pituitary stalk (PS). Stray voxels were isolated and removed from all labels. Average aneurysm wall thickness was measured on source imaging using the Picture Archiving Communication System (Carestream Vue PACS, Rochester, NY). The aneurysm wall thickness was conservatively measured on T1 + Gd imaging in the sagittal, coronal, and axial planes using a slice that cut through the largest cross section of the aneurysm that allowed for the best visualization of the wall. Measurements were taken at the thinnest and thickest sections of the wall that could be visualized in each view, and subsequently averaged. (See Supplementary Fig. S5). This procedure was conducted by two investigators, wherein disagreement in thickness of more than 0.25 mm difference was adjudicated by an experienced and senior investigator. Additionally, mural thrombosis was identified as high T1 signal, or isointense signal to the normal vessel wall.

### Semi-automatic SI quantification and 3D AWE reconstruction

The SI of the aneurysm wall was mapped from T1 and T1 + Gd images using image processing tools and custom scripts in MATLAB 2020a (The MathWorks, Natick, MA). Base images were isotropically resampled using a cubic interpolation method. A bounding box was created with a 25 mm radius from the aneurysm and vessel labels. Labels were smoothed using a 3 mm gaussian kernel with a 1 mm standard deviation (SD). Larger aneurysms were smoothed with a 5 mm or 7 mm kernel depending on size. Isosurfaces were created from the smoothed and interpolated labels. Surface complexity was reduced to 1 or 10% of the original surface for medium and large aneurysms. Small aneurysms (< 5 mm) maintained their original surface complexities.

**Figure 8:**
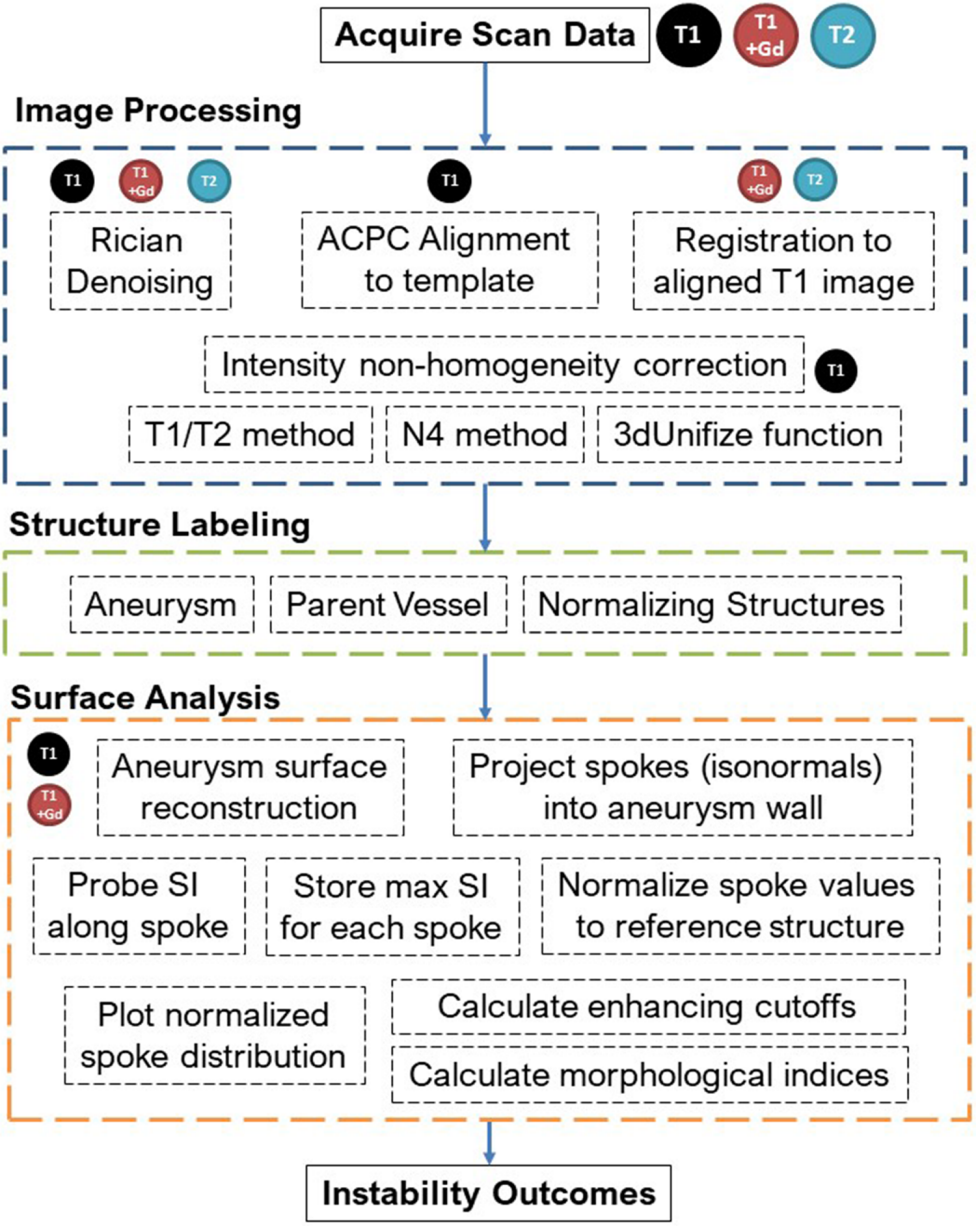
Flow chart depicting semi-automated reconstruction and quantification of AWE from image acquisition to final analysis.

For each vertex in the aneurysm surface model, isonormals were calculated, creating spokes extending outward from the previously generated masks at a distance equivalent to the average aneurysm wall thickness. Parent artery labels were used to exclude spokes created at the neck of the aneurysm. Along each spoke, the SI from the base image was calculated using a cubic spline interpolation. The maximal value of each spoke was used to determine the SI of the wall and to eliminate luminal contamination and potential errors from radiological wall thickness measurements. The raw SI values for each spoke were normalized to the max SI values of the CC and PS. 3D heatmaps were created by superimposing the SI data on the outer aneurysm surface as determined by the outermost vertex of each spoke. AWE was calculated as the mean value of each spoke (Fig. 8).

### Semi-automatic bleb analysis

Blebs were identified based on criteria defined by Ashkezari et al.^44^ Average bleb wall thickness was calculated as the average of five measurements in the cardinal planes. Blebs were then manually labeled on the processed T1 + Gd images according to the same protocol described for aneurysms. Bleb raw values were also normalized to the CC and PS.

### Manual SI quantification

Manual SI ROIs were sampled to compare the semiautomated 3D mapping method with the manual method of determining AWE. The manual method has been used extensively by our group and other groups to quantify AWE^7,8^. Wall enhancement of each aneurysm was measured with PACS. AWE was quantified by creating ROIs of the aneurysm wall in each plane on source T1 + Gd sequences as previously described^7,45^. ROIs were sampled in three different planes and after co-registration of the T1 + Gd and T1 sequences. Normalization to the PS or CC was calculated as follows: (Mean or Max SI_wall post_)/(Mean or Max SI_PS_ or SI_CC_). Similarly, bleb ROIs were used to manually determine wall enhancement, and normalized to the PS and CC.

### Morphological indices

Planar-isolated aneurysm segmentations were created on T1 + Gd aneurysm labels using 3D Slicer. These labels were processed with source images using MATLAB to find label volume, surface area, convex hull volume, and convex hull surface area. Ellipticity index (EI), non-sphericity index (NSI), and undulation index (UI) were subsequently calculated for each aneurysm according to Raghavan et al.^46^ Aneurysm size, size and aspect ratios were manually calculated from subtraction angiograms, computed angiograms or magnetic resonance angiograms as described by Dhar et al.^16^.

### Statistical analysis

All statistical analysis was conducted using SAS (SAS Institute, Cary, North Carolina) and SPSS Statistics 25 (IBM, New York, USA). Categorical variables are presented as frequency and percentage, and continuous variables as mean ± SD. A Student t-test was used to compare continuous data, and Pearson chi-squared test for the relationship between categorical data. The differences of manual measurements of aneurysm size, aspect and size ratios, and wall thickness were analyzed with Bland–Altman plots. The manual and semi-automated methods for measuring SI were compared for agreement using Pearson’s correlation. Bleb SI was compared to aneurysm SI using the Wilcoxon Signed Rank Test^47^. Logistic regression analysis was used to determine the association of aneurysm instability based on size > 7 mm^18^ with morphological and AWE predictors. Odds ratios (ORs) and 95% confidence intervals were constructed for each predictor. A receiver operating characteristic (ROC) analysis was performed with cutoffs calculated according to the Youden Index^48,49^. All statistical tests used two-tail alternatives and assessed significance at α = 0.05.

## Supplementary Information

Supplementary Figures.

## Data Availability

The data analyzed in this study are available from the corresponding author upon reasonable request.

## Code availability

The code developed for this study is available from the corresponding author upon reasonable request.
