# Supplementary material for: Semiautomated 3D mapping of aneurysmal wall enhancement with 7T-MRI
Source: Sci Rep. 2021 Sep 15;11:18344. doi: 10.1038/s41598-021-97727-0 (PMC8443635; doi:10.1038/s41598-021-97727-0)
Supplement: Supplementary file 1 — Supplementary Figures. [file 41598_2021_97727_MOESM1_ESM.pdf]

Supplementary Information

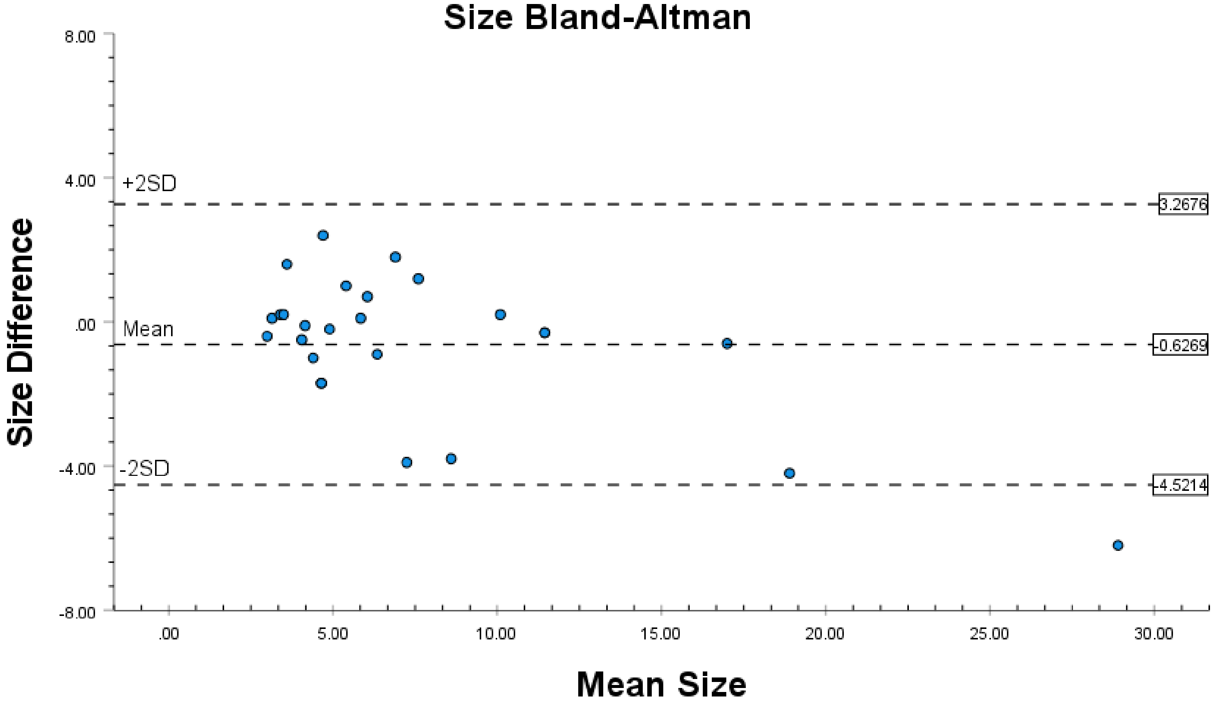

Supplementary Figure S1. Bland-Altman plot of aneurysm size measurements between two independent raters

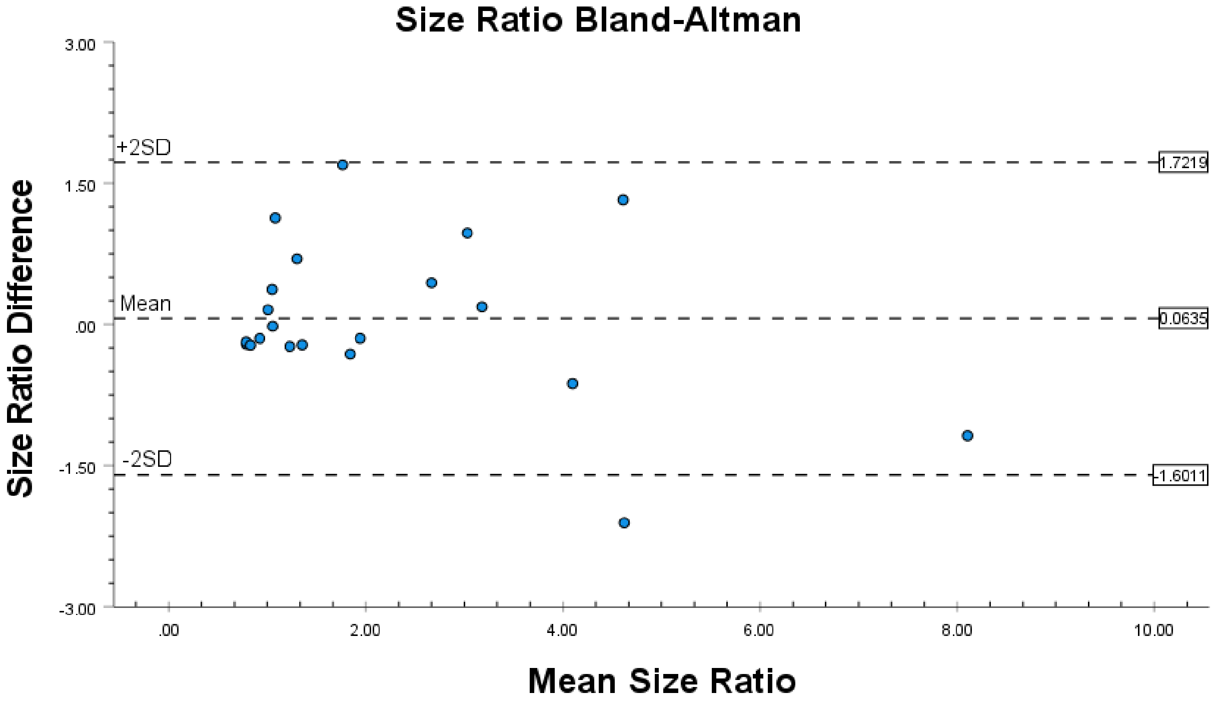

Supplementary Figure S2. Bland-Altman plot of aneurysm size ratio measurements between two independent raters

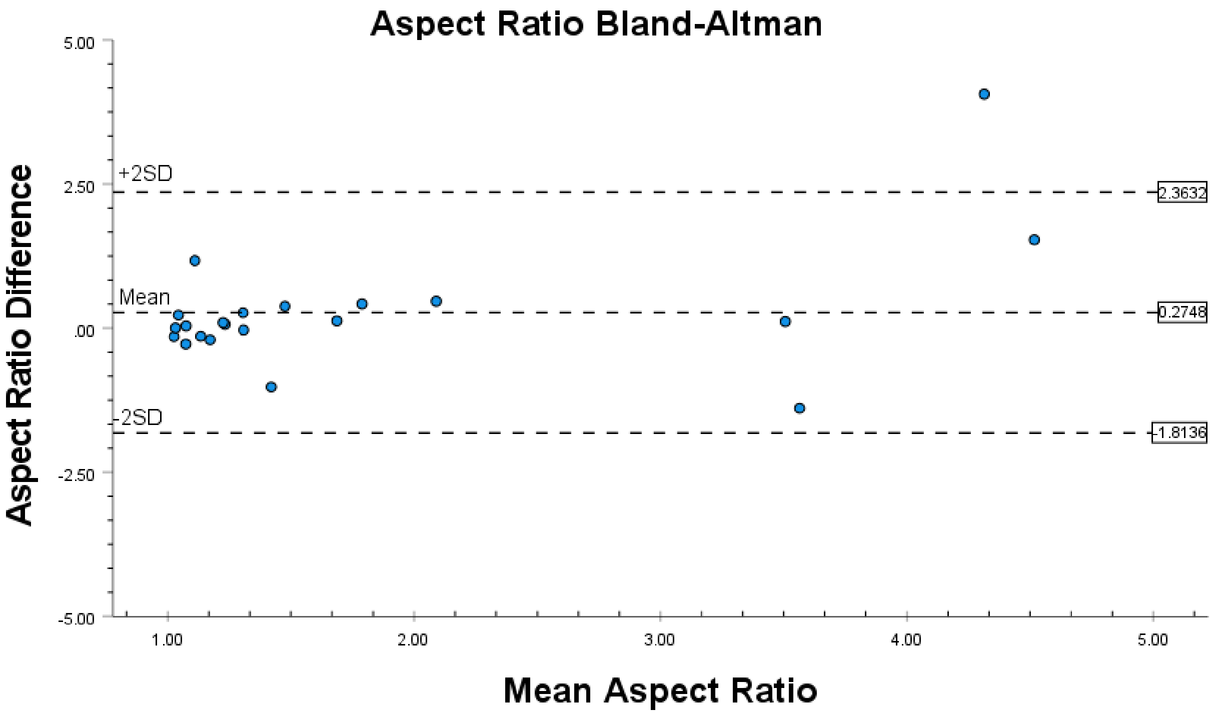

Supplementary Figure S3. Bland-Altman plot of aneurysm aspect ratio measurements between two independent raters

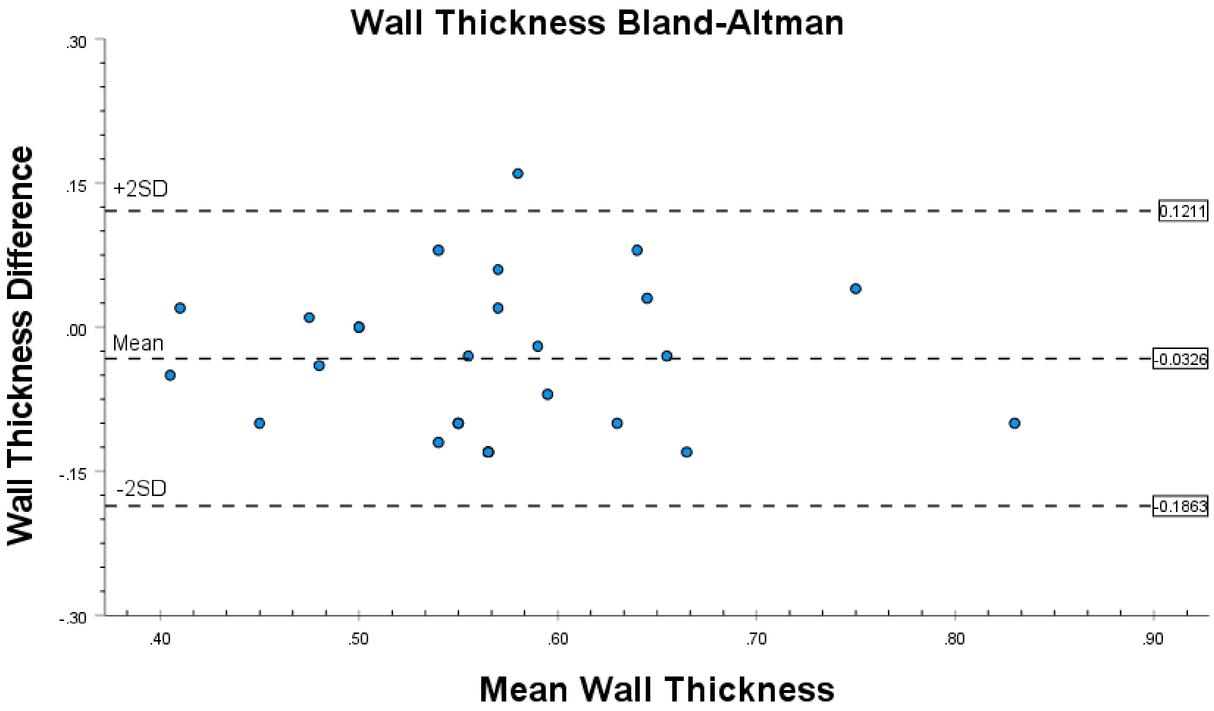

Supplementary Figure S4. Bland-Altman plot of aneurysm wall thickness measurements between two independent raters

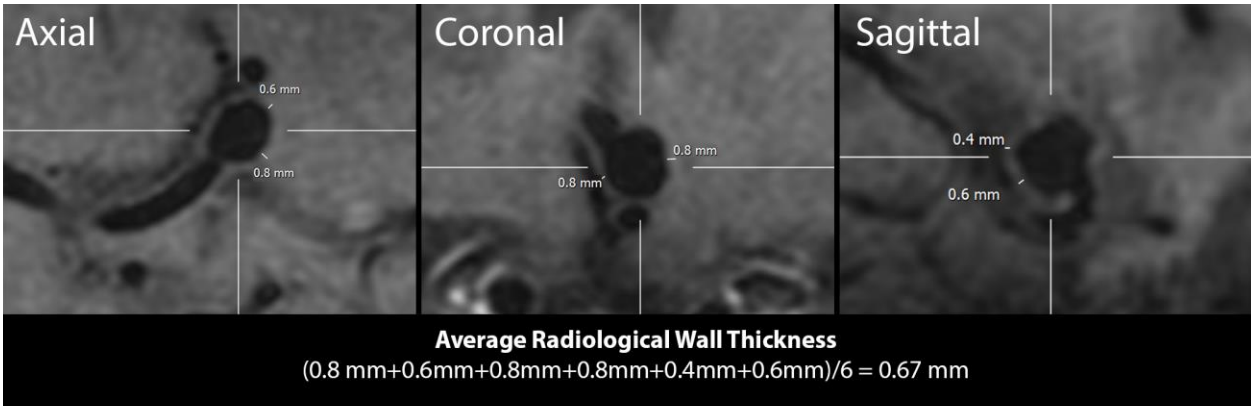

Supplementary Figure S5. Wall thickness measurements for an ACOM aneurysm taken in the axial(left), coronal(middle), and sagittal (right) views show the thickest and thinnest sections of the wall in each view, with an average wall thickness of 0.67 mm.
